# Metabolomic and lipidomic profiling of the spinal cord in type 2 diabetes mellitus rats with painful neuropathy

**DOI:** 10.1007/s11011-024-01376-x

**Published:** 2024-07-09

**Authors:** Zhuoying Yu, Jing Yang, Ye Jiang, Min Wei, Yanhan Lyu, Dongsheng Yang, Shixiong Shen, Yongzheng Han, Min Li

**Affiliations:** Department of Anesthesiology, Peking University Third Hospital, Beijing, 100191 China

**Keywords:** Painful diabetic neuropathy, Metabolomic, Lipidomic, Spinal cord

## Abstract

In this paper we investigated lipid and metabolite changes in diabetic neuropathy, using untargeted lipidomics and metabolomics analyses of the spinal cords from streptozotocin-treated diabetic rats.170 metabolites and 45 lipids were dysregulated in the painful diabetic neuropathy (PDN) phase. Pathway enrichment analysis revealed perturbations in starch and sucrose, tryptophan, pyrimidine, cysteine and methionine, thiamine, tyrosine, and nucleotides. The disturbance of tyrosine, tryptophan, methionine, triacylglycerol, and phosphatidylethanolamine metabolism indicated that pathological mechanisms in the PDN involved energy metabolism, oxidative stress, and neural reparative regeneration. These revelations offered potential biomarkers for PDN and enriched the comprehension of the complex molecular mechanisms characterizing PDN, establishing a solid foundation for subsequent inquiries into neural convalescence and recovery after PDN.

**Supplementary Information:**

The online version contains supplementary material available at 10.1007/s11011-024-01376-x.

## Introduction

Painful diabetic neuropathy (PDN) refers to the occurrence of pain in patients due to peripheral nerve damage caused by diabetes mellitus (DM) or prediabetes. The reported incidence of PDN varies in different studies, attributed to factors such as patient selection bias and diagnostic method discrepancies. The estimated prevalence rate of diabetic neuropathy ranges from 6 to 51% in patients with DM or prediabetes (Sloan et al. 2021). Among these individuals, 30–50% may progress to PDN (Feldman et al. 2019). In clinical practice, PDN often manifests as symmetric peripheral neuropathic pain predominantly affecting the distal extremities with mononeuropathy or radicular pain in the brachial or lumbosacral plexus. Patients commonly experience moderate to severe stabbing, burning, sharp, shooting, tearing sensations, and even electric shock sensations. PDN is a significant contributor to increased mortality and disability rates among DM patients, and it poses complex care and medical challenges, greatly impacting patients’ sleep, mood, and quality of life (Pop-Busui et al. 2017). Therefore, developing proactive and effective prevention and treatment strategies is crucial for early intervention in PDN.

While the underlying mechanisms of PDN are not yet fully understood, there is a prevailing consensus that hyperglycemia-mediated cellular detriment is a key factor in its pathogenesis (Braffett et al. 2020; Feldman et al. 2019; Jeyam et al. 2020). Emphasis is placed on the nexus between PDN and heightened glucose metabolism (Braffett et al. 2020; Callaghan et al. 2012), which triggers the activation of the polyol, hexosamine, and protein kinase C pathways (Brownlee 2001), leading to the formation of advanced glycation end products and their receptors (Khalid et al. 2022; Nowotny et al. 2015). Additionally, in type 1 DM, insulin deficiency leads to impaired insulin signaling, while in type 2 DM, insulin resistance results in weakened PI3K-AKT signaling (Camaya et al. 2022; Huang et al. 2018). Dyslipidemia and hyperglycemia, in tandem, instigate inflammatory cascades that lead to microvascular dysfunction, DNA degradation, endoplasmic reticulum stress, and mitochondrial perturbation, ultimately culminating in neuronal demise (Cox et al. 2017; Eftekharpour and Fernyhough 2022; Fernyhough 2015; Fernyhough et al. 2010; Patel et al. 2023). A deeper investigation into the intricate mechanisms of PDN holds significant importance for its prevention and improvement of life quality in PDN patients.

As a prototypical metabolic disorder, DM disrupts glucose metabolism and affects the metabolism of amino acids, lipids, purines, and other substances. This disruption can cause metabolic disturbances, accelerating the progression of PDN. Therefore, it is crucial to understand PDN from a metabolic perspective. Metabolomics is commonly defined as the complete collection of metabolites, or small molecule chemicals, found in a given organelle, cell, organ, biofluid, or organism (Wishart 2019). Metabolomics typically encompasses both targeted and untargeted approaches to measure metabolic changes. Targeted metabolomics studies focus on a known set of specific metabolites, whereas untargeted metabolomics aims to capture all the metabolites present in a sample (Szeremeta et al. 2021). Due to the limited coverage of targeted approaches, a non-targeted metabolomics approach was selected in this study. In DM research, metabolomics has been used to identify biomarkers for type 2 DM (Arneth et al. 2019; Jin and Ma 2021) and its attendant complications, while also exploring options for pharmacological intervention (Jin and Ma 2021). Therefore, this study aims to examine the complexities of PDN through metabolomic and lipidomic analyses, with a particular focus on metabolite scrutiny.

The spinal cord, particularly its dorsal horn, plays a critical role in processing and transmitting sensory information related to pain (Todd 2010). Diabetic neuropathic pain involves pathological changes not only in peripheral nerves but also within the central nervous system, including the spinal cord (Tsuda 2016). Sampling tissue from the spinal cord allows direct access to the site where biochemical and metabolic changes associated with neuropathic pain occur. We hypothesized unique metabolites and lipids within the spinal cord were intricately linked to PDN in type 2 DM rats. Through the meticulous application of metabolomics, lipidomics, and bioinformatics analyses on the spinal cord, we aimed to systematically elucidate the dysregulation of microenvironmental metabolism. Our findings provide valuable scientific clues for the investigation of PND neuropathogenesis and facilitate more specific biomarker studies in the field. 

## Materials and methods

### Animals and groups

This study involved 38 specific pathogen-free Sprague-Dawley rats, carefully selected with body weights ranging from 157 to 189 g. These rats were obtained from the Experimental Animal Center of Peking University Health Science Center (approval number: PUIRB-LA2022638). The implementation of these animal trials rigorously adhered to the principles articulated in the ARRIVE guidelines and the esteemed precepts outlined in the National Research Council’s Guide for the Care and Use of Laboratory Animals. Rats were housed within the confines of individually ventilated cages, maintained under standard conditions, and subjected to a 12-hour light/dark cycle The rats were randomly allocated into two cohorts: the PDN (*n* = 19) and the control group (*n* = 19).

### Type 2 DM modeling

The establishment of type 2 DM rats was conducted following the methods described in the literature (Dang et al. 2014). High-fat diet (containing 45% fat, 20% protein, and 35% carbohydrates) sustained over 8 weeks, the rats subsequently were fasted for 12 h. DM was induced by administering streptozotocin (STZ) via a single intraperitoneal injection of 30 mg/kg, meticulously prepared in a citrate-sodium citrate buffer. 3 days after the STZ injection, the fasting blood glucose levels ≥ 11.1 mmol/L confirmed the successful establishment of the DM model (Zhou et al. 2017). In contradistinction, the control group was injected with an equal volume of citrate-sodium citrate buffer.

### Intraperitoneal glucose tolerance and insulin tolerance experiments

1. Intraperitoneal glucose tolerance test (IPGTT)
  Each group consisted of 11 rats, all of which were subjected to an IPGTT 1 week after STZ injection intraperitoneally. The rats were fasted overnight for 12 h, with access to water, and then intraperitoneally injected with a 50% glucose solution (2 g/kg). Blood glucose was measured before the injection and subsequently at 30, 60, and 120 min (Gou et al. 2021).
2. Insulin tolerance test (ITT)
  A fortnight after the STZ injection, each group of 11 rats underwent an ITT. An intraperitoneal administration of regular insulin (NovoRapid, Novo Nordisk, Denmark) at a dosage of 0.5 U/kg was executed. Blood glucose was measured before the injection and subsequently at 30, 60, and 120 min. The ITT evaluation was based on the relative decrease in serum glucose compared with the baseline level (Saande et al. 2019).

To validate the occurrence of neuropathic pain in type 2 DM rats, a von Frey and a hot plate test were conducted at the beginning of the experiment and on days 7, 14, 21, 28, and 35 after STZ injection. Evaluators of all behavioral experiments were blinded to the group assignments.

### Assessment of mechanical allodynia

Based on our previous research (Yang et al. 2024), mechanical pain was assessed by measuring the 50% paw withdrawal threshold (PWT). Before testing, the rats were placed in an organic glass cylinder for sufficient adaptation. A series of standardized Von Frey filaments were then used to stimulate the plantar surface of the rat’s paw, and the rapid withdrawal response within the stimulation time was recorded as a positive response. Eight Von Frey filaments were used with forces of 0.41, 0.70, 1.20, 2.00, 3.63, 5.50, 8.50, and 15.10 g. The test began with a filament of moderate force (2.00 g) and followed the “up and down” method (Chaplan et al. 1994).

### Assessment of thermal hyperalgesia

Following the previous study, a test was conducted to assess thermal hyperalgesia in the hind limbs (Yang et al. 2024). Rats were placed on a transparent glass plate and were allowed at least 30 min to acclimate within the cage. The plantar surface of the hind paw served as the target for the radiant heat source. Activation of the heat source initiated a timer, measuring the paw withdrawal latency (PWL), which ceased upon detection of paw withdrawal. The average of three PWL measurements from each hind limb was recorded as the outcome.

### Sensory nerve conduction velocity (SNCV)

Based on previous studies (Baum et al. 2016; Davidson et al. 2018), this method for measuring SNCV involved using the digital nerve of the second toe, placing recording electrodes at the sciatic notch, and positioning stimulating electrodes near the ankle to deliver electrical stimulation. SNCV was calculated by dividing the distance between the stimulating and recording electrodes by the latency period of the initial positive peak, with the resulting value expressed in meters per second.

### Spinal cord tissue collection

After anesthesia was administered, the rats were securely fixed onto a surgical board. The heart was exposed and rapidly perfused with saline. Upon completion of perfusion, the spinal cord was extracted by delicately dissecting the spinal canal in a cranial direction, starting from the sacrum, using tissue scissors and bone forceps. This technique allowed for the retrieval of the L4-5 spinal cord segment.

### Transmission electron microscopy

To perform spinal cord electron microscopy, following tissue collection, spinal cord samples were fixed in a 2.5% glutaraldehyde solution in 0.1 M sodium bicarbonate buffer for 24 h. The fixed spinal cord was then embedded in Epon resin to produce 70-nm-thick sections (Li et al. 2023). The segments were stained with uranyl acetate and lead citrate, and the ultrastructure of the spinal cord was examined using the Japanese JEM-1400 transmission electron microscope (Japan Electron Optics Laboratory Co., Tokyo, Japan). Images were captured at a magnification of 10,000×. To analyze the spinal cord ultrastructure, 20 random fields were selected from each group, with three rats per group.

### Sample preparation

The extracted spinal cord was rapidly frozen and then, 30 mg spinal cord was retrieved and placed into a 2 mL centrifuge tube. Subsequently, 900 µL of extraction solution (methanol: water = 4:1) was employed for metabolite extraction. Samples of spinal cord were ground in a cold tissue grinder for 6 min (-10℃, 50 Hz), followed by low-temperature ultrasonic extraction for 30 min (5℃, 40 kHz). The samples were then left at -20℃ for 30 min, centrifuged for 5 min (4℃, 12,000 ppm), and the supernatant was transferred to sample vials equipped with insert tubes for subsequent analysis.

### Quality control (QC) sample

As a part of the system conditioning and quality control process, a quality control sample was prepared by mixing equal volumes of all samples. A stringent QC regimen was observed to safeguard the reliability of the acquired data. This regimen entailed the equitable amalgamation of spinal cord samples, ensuring uniform representation from all specimens. These QC samples were inserted into the analytical sequence between every five analyzed samples throughout the metabolomic and lipidomic investigations.

### Liquid chromatography mass spectrometry

Data-dependent acquisition (DDA) was employed utilizing a Q-Exactive HF mass spectrometer (Thermo Fisher Scientific, Waltham, Massachusetts, USA) in strict adherence to time-honored protocols (Zhou et al. 2021). The inception of the acquisition cycle unfolds with a solitary survey scan (MS1) executed at a resolution of 60,000. This sweeping scan encapsulated the expanse of the mass-to-charge ratio (m/z) spectrum, spanning from 60 to 900 for hydrophilic metabolites and from 300 to 1200 m/z for the ethereal realm of lipids. Then, a sequence of 10 MS/MS scans was conducted in high-energy collisional dissociation mode to collect various types of molecular information. 

### DDA-MS data analysis

The raw data obtained through DDA-MS analysis underwent processing using MS-DIAL software version 3.60, following the guidelines of the user manual, as previously described (Zhou et al. 2021). In brief, metabolite identification, based on both MS1 and MS2 spectra, was conducted within MS-DIAL. Acquired spectra underwent scrutiny against the MassBank database seamlessly integrated within the sophisticated MS-DIAL software, which comprised a staggering 8068 entries in the realm of positive electrospray ionization, complemented by 4782 entries in the domain of negative electrospray ionization. In the process of lipid identification, MS-DIAL judiciously leveraged the LipidBlast - derived in silico spectra database (version: LipidDBs-VS23-FiehnO). The parameters governing the scrutiny of MS1 and MS/MS spectra were marked by exacting tolerances, standing at 0.01 Da and 0.05 Da, respectively, all while adhering to a discerning identification scoring threshold set at 70%. Additional facets of the MS-DIAL analysis remained steadfast at their default configurations.

### Statistical analysis

The assessment of metabolic profiles and distinctions between the PDN cohort and the control ensemble underwent scrutiny through the prism of principal component analysis (PCA) and partial least squares discriminant analysis (PLS-DA). Criteria for the identification of differentially expressed metabolites included a variable importance in projection (VIP) > 1 in the PLS-DA model, coupled with a *P*-value < 0.05 in the t-test. The analytical prowess of MetaboAnalyst 5.0 (https://www.metaboanalyst.ca), a cybernetic bastion, was harnessed for PCA, PLS-DA, and pathway enrichment analysis, with the venerable Kyoto Encyclopedia of Genes and Genomes database standing as a fount of reference.

In the quest for an all-encompassing vista of metabolic intricacies, the spinal cords of both the control and PDN-afflicted rats underwent meticulous analysis through the prism of MS in both positive and negative ion modalities. PCA and PLS-DA, those discerning arbiters of visualizing general distribution patterns, were summoned forth to unveil the overarching tapestry of the spinal cord samples.

For the data analysis, the SPSS software (version 26.0) presented results as mean ± standard error of the mean (SEM). The normal distribution of the data was evaluated through the application of the Shapiro-Wilk test, while an independent samples t-test was employed for comparing two groups, and while two-way ANOVA test was applied to compare multiple groups, as appropriate. Additionally, the examination of the correlation between two variables was conducted using Spearman’s rank correlation analysis. For the significance of correlation differences, the Benjamini-Hochberg method was used to calculate the adjusted *P* values, with *P* values < 0.05 considered statistically significant.

## Results

### PDN rat characteristics

At the beginning of the research, rats had an initial body weight in the range of 157-189 g, and over 8 weeks, their body weights consistently increased.

**Fig. 1:**
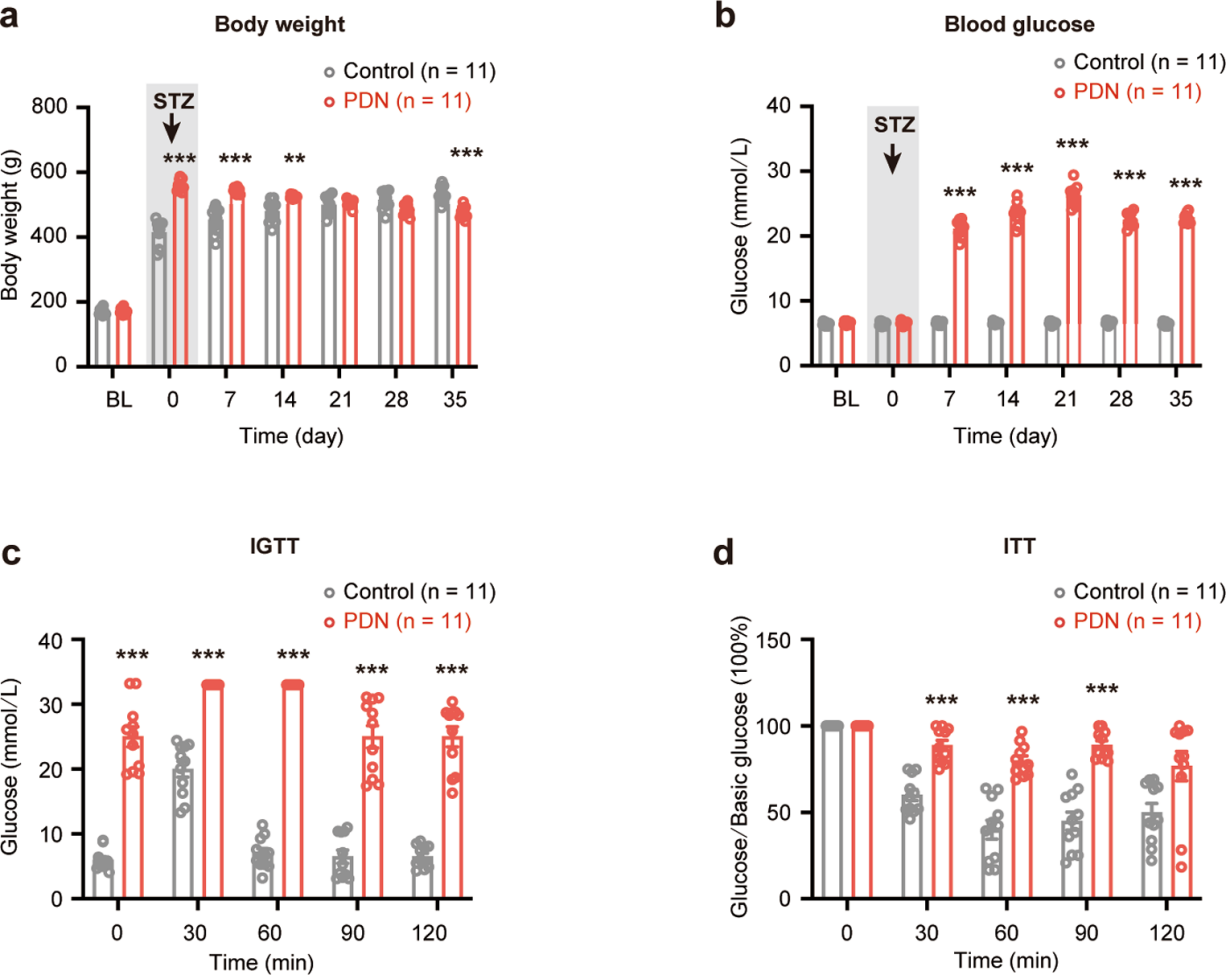
Changes in the body weight, blood glucose level, intraperitoneal glucose tolerance test (IPGTT), and insulin tolerance test (ITT) results in type 2 painful diabetic neuropathy (PDN) rats. **a** and **b** PDN rats exhibited significant alterations in body weight and blood glucose levels over time compared with those in the control group (**P < 0.01 and ***P < 0.001, respectively; *n* = 11); **c** An IPGTT on the 7th day after streptozotocin (STZ) injection revealed a significant difference in PDN rats (***P < 0.001; *n* = 11);**d** An ITT on the 14th day after STZ injection demonstrated a significant response compared with the control group (***P < 0.001; *n* = 11); data are presented as mean ± SEM;  two-way ANOVA with Sidak’s multiple comparisons tests for **a**-**d**

By the end of the eighth week, the PDN group exhibited a significant increase in body weight compared to the control group. However, following STZ administration, the body weight of the PDN group decreased (Fig. 1a).

One week following STZ injection, the PDN group demonstrated a significant elevation in blood glucose levels (Fig. 1b). IPGTT and ITT were conducted to examine impaired glucose control within the PDN group. Following glucose administration, blood glucose levels in the PDN group exhibited a significant increase, which persisted from 30 to 60 min. Moreover, the PDN group displayed more pronounced fluctuations in blood glucose levels in response to insulin administration than those in the control group (Fig. 1c and d). 

**Fig. 2:**
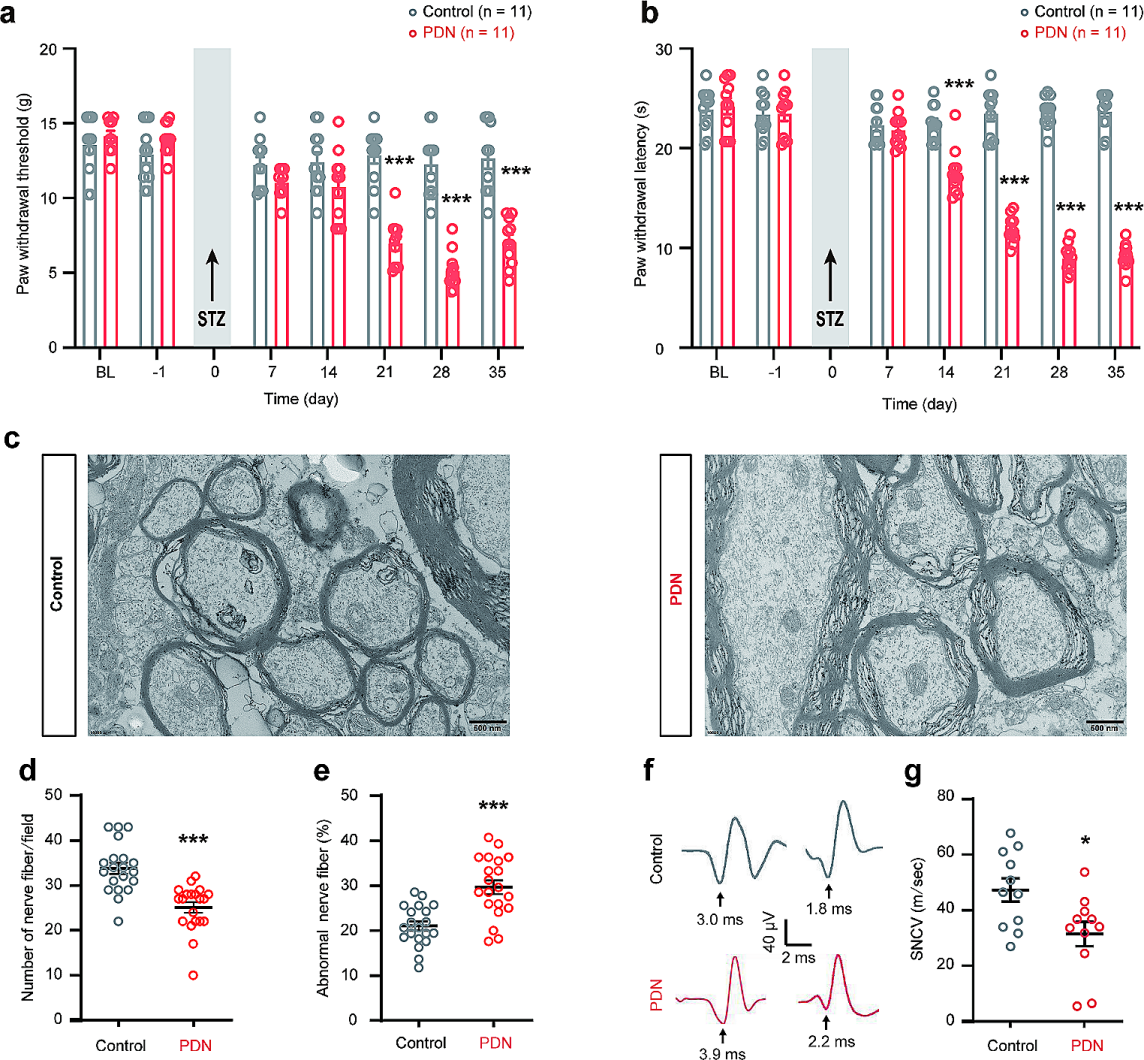
Confirmation of type 2 diabetic neuropathy in rats. **a** Paw withdrawal threshold (PWT) of rats after streptozotocin (STZ) injection (*** *P* < 0.001, *n*= 11); **b** Paw withdrawal latency (PWL) of rats after STZ injection (*** P< 0.001, *n* = 11); **c** Representative electron micrographs of the spinal cord;**d** Number of nerve fibers per field in the spinal cord (***P < 0.001,*n* = 3); **e** Number of abnormal nerve fibers per field in the spinal cord (***P< 0.001, *n* = 3); **f**, **g** sensory nerve conduction velocity (SNCV) of rats following STZ injection (*P < 0.05, *n* = 11); data are presented as mean ± SEM; two-way ANOVA with Sidak’s multiple comparisons tests for **a**-**b**, unpaired t-tests (two-tailed) for**d**, **e**, and **g**

With regard to baseline pain and type 2 DM-related pain, 21 days after STZ injection, the PDN group of rats exhibited a significant decrease in the PWT (Fig. 2a). Similarly, 14 days post-STZ injection, the PDN group demonstrated heightened sensitivity to thermal stimulation (Fig. 2b), signifying a manifestation of abnormal pain behavior. Considering the prior findings, we utilized the time point of 21 days post-STZ injection as the basis for subsequent specimen collection.

Ultrastructural features of myelinated axons were examined with electron microscopy, and myelination was manually counted. The control group exhibited profuse myelinated axons with densely packed loops of myelin enveloping the axons. In contrast, pronounced regions of demyelination and extensive axonal degeneration, characterized by full-thickness demyelination, were observed within the PDN rats. Additionally, myelination became noticeably less abundant, accompanied by a substantial increase in the number of damaged axons, in the PDN group (Fig. 2c-e). These findings collectively indicated a significant reduction in axon density and noteworthy structural impairment of axons in PDN rats.

Measurement of SNCV has become a pivotal endpoint in both preclinical and clinical research. Thus, we further analyzed the SNCV in PDN rats. The PDN group exhibited a reduction of approximately 45% in sciatic nerve SNCV (Fig. 2f and g). These experimental findings collectively suggested the progression of PDN in type 2 DM rats.

### Untargeted metabolic and lipidomic profiles of the spinal cord

**Fig. 3:**
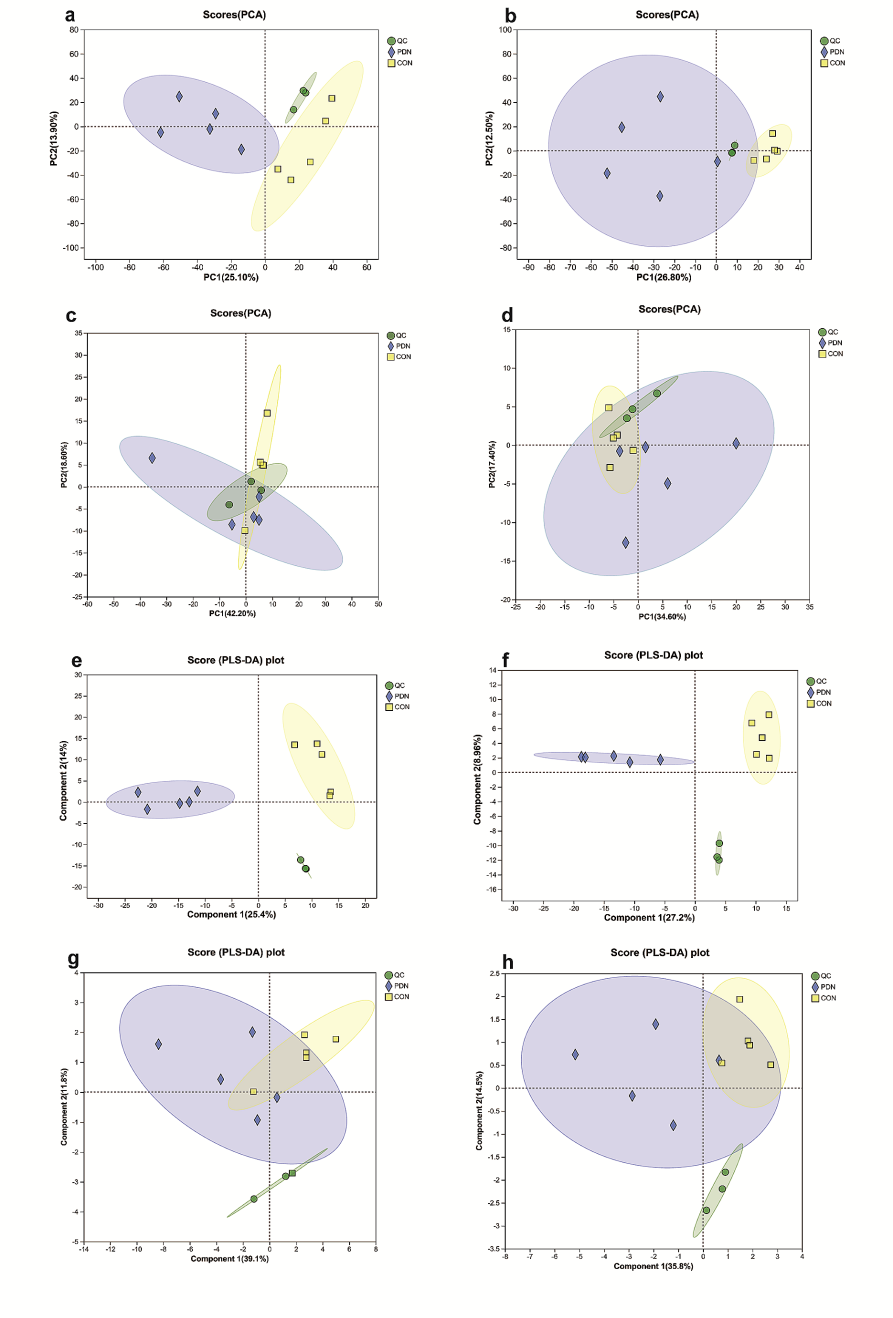
Principal component analysis (PCA) score plots and Partial least squares discriminant analysis (PLS-DA) score plots of hydrophilic and lipophilic substances in spinal cord samples of both control and PDN rats. PCA (**a** Positive ion mode of hydrophilic substances; **b** Negative ion mode of hydrophilic substances; **c** Positive ion mode of lipophilic substances; **d** Negative ion mode of lipophilic substances); PLS-DA (**e** Positive ion mode of hydrophilic substances; **f** Negative ion mode of hydrophilic substances; **g** Positive ion mode of lipophilic substances; **h** Negative ion mode of lipophilic substances). The groups are indicated by different colors (green: quality control (QC); blue: PDN; yellow: control)

The PCA score plot demonstrated a separation trend in terms of hydrophilic and lipophilic substances in both the positive and negative ion modes, indicating significant differences in the metabolic profiles of PDN rats compared with those of normal rats (Fig. 3a and d). The PLS-DA score plot further indicated intergroup differences in the metabolic profiles of the spinal cords (Fig. 3e and h).

**Fig. 4:**
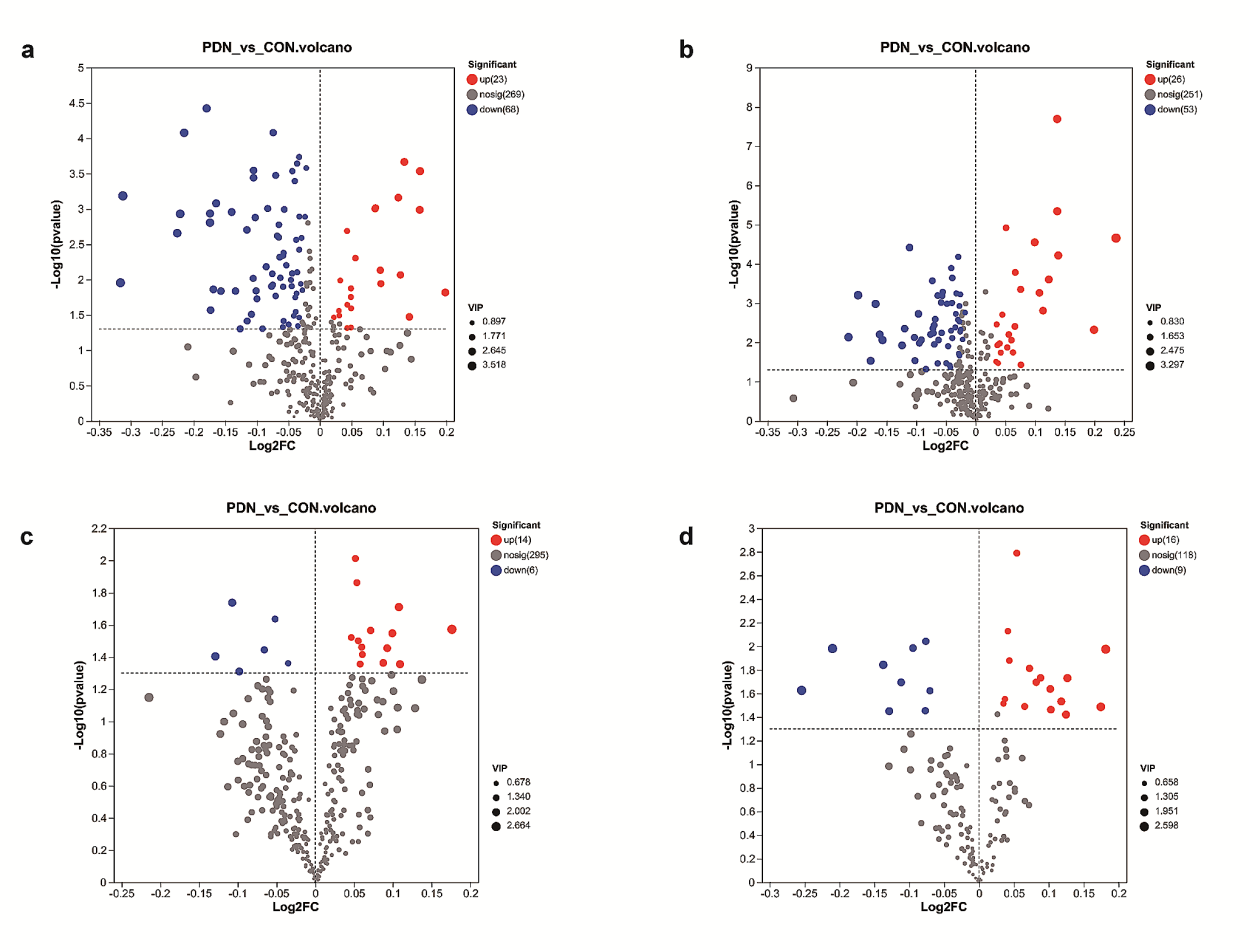
Volcano plot of hydrophilic and lipid metabolites in both positive and negative ion mode. **a** Positive ion mode of hydrophilic substances; **b** Negative ion mode of hydrophilic substances; **c** Positive ion mode of lipophilic substances; **d** Negative ion mode of lipophilic substances. Every datum on this graphical tableau aligns with a metabolite, where the red unveils the significantly upregulated entities, the blue unveils the significantly downregulated counterparts, and the grey unveils those metabolites that remain non-significantly different to the normal group. The classification is based on a VIP > 1 and a significance level of *P*< 0.05

Upon identifying the metabolic differences in PDN rats, further screening of differential metabolites between the two groups was conducted using the PLS-DA method. In the realm of hydrophilic metabolites, 360 features showed in positive ion mode, accompanied by 330 in the realm of negative ion mode. As for lipid metabolites, 315 features displayed in positive ion mode, while 143 executed in negative ion mode. A subsequent foray into statistical discourse unveiled 170 perturbed hydrophilic metabolites and 45 perturbed lipid metabolites discerned between the realms of PDN and the ethereal control ensemble (VIP > 1 and *P* < 0.05). The PDN group exhibited upregulation of 49 hydrophilic metabolites significantly different from the control, along with downregulation of 121 hydrophilic metabolites (Fig. 4a and b). Moreover, 30 lipid metabolites were upregulated and 15 hydrophilic metabolites were downregulated (Fig. 4c and d). The top 20 dysregulated hydrophilic (Table 1) and lipid metabolites (Table 2) in PDN rats, based on the descending order of the *P* value, were presented in the tables below. Differential metabolites were further screened with a false discovery rate < 0.05 and VIP > 1. The results indicated that there were changes in 72 hydrophilic metabolites between the PDN and control groups (Fig. S1), while no significant differences were observed in the lipophilic substances.

**Table 1:** The top 20 differentiating metabolites between the PDN and control groups identified from the hydrophilic substances

Metabolites	VIP	*P*-value	FC (*P*/C)	Trend
D-Glucose 1-phosphate	2.55	2.03E-08	1.10	up
L-Iditol	2.49	4.55E-06	1.10	up
Propionic acid	1.64	1.20E-05	1.04	up
Dulcitol	2.43	2.82E-05	1.07	up
Glu-Thr	2.15	3.83E-05	0.93	down
L-Threonine	1.33	6.59E-05	0.98	down
5,6-Dihydro-5-methyluracil	2.11	8.32E-05	0.95	down
Cocaethylene	2.92	8.35E-05	0.86	down
Mefenamic acid	1.36	1.28E-04	0.97	down
16-Hydroxyhexadecanoic acid	1.69	1.65E-04	1.05	up
L-Anserine	1.40	1.84E-04	0.98	down
Rosmarinic acid	2.47	2.16E-04	1.10	up
4-Guanidinobutyric acid	1.50	2.28E-04	0.98	down
Pyroglutamic acid	1.54	2.28E-04	0.97	down
L-(+)-Rhamnose	2.32	2.50E-04	1.09	up
N-Methylproline	1.18	2.63E-04	0.99	down
Allocryptopine	2.22	2.85E-04	0.93	down
Thiamine monophosphate	1.47	2.92E-04	0.97	down
1-(3-Chlorophenyl)piperazine	1.90	3.35E-04	0.95	down
3-Hydroxy-beta-lapachone	2.23	3.62E-04	0.93	down

*VIP*, variable importance in projection; *FC* fold change

**Table 2:** The top 20 differentiating lipids between the PDN and control groups

Lipids	VIP	*P*-value	FC(*P*/C)	Trend
SL(49:7O)	1.33	1.62E-03	1.04	up
PE(O-16:1/20:3)	1.13	7.43E-03	1.03	up
PC(18:1/16:1O)	1.29	9.08E-03	0.95	down
TG(O-15:0/16:0/16:0)	1.14	9.72E-03	1.04	up
PC(24:0/18:1)	1.45	1.03E-02	0.94	down
Cer(18:02O/24:1)	2.12	1.05E-02	0.86	down
TG(16:1/18:2/18:3)	1.99	1.06E-02	1.13	up
PE(18:0/22:5)	1.09	1.32E-02	1.03	up
TG(O-18:0/16:0/18:0)	1.21	1.37E-02	1.04	up
PC(O-16:0/20:4)	1.69	1.43E-02	0.91	down
TG(18:1/20:1/18:2)	1.27	1.54E-02	1.05	up
PC(O-38:4)	1.57	1.83E-02	0.93	down
SHexCer(35:13O)	1.38	1.85E-02	1.06	up
TG(16:0/18:2/18:3)	1.62	1.86E-02	1.09	up
DG(52:6)	1.46	1.95E-02	1.08	up
TG(18:2/18:3/18:3)	1.30	2.02E-02	1.06	up
Cer(18:02O/22:0)	1.48	2.02E-02	0.93	down
TG(16:0/18:1/22:4)	1.47	2.30E-02	1.07	up
Cer(18:12O/26:0)	1.03	2.31E-02	0.96	down
PC(O-46:12)	2.51	2.36E-02	0.84	down

*VIP*, variable importance in projection; *FC*, fold change; *SL*, saccharolipid; *TG*, triacylglycerol; *PC*, phosphatidylcholine; *PE*, phosphatidylethanolamine; *Cer*, ceramide; *SHexCer*, sulfated hexosylceramide; *DG*, diacylglycerol; *HexCer*, hexose ceramide

### Bioinformatic analysis revealed perturbed metabolic pathways

**Fig. 5:**
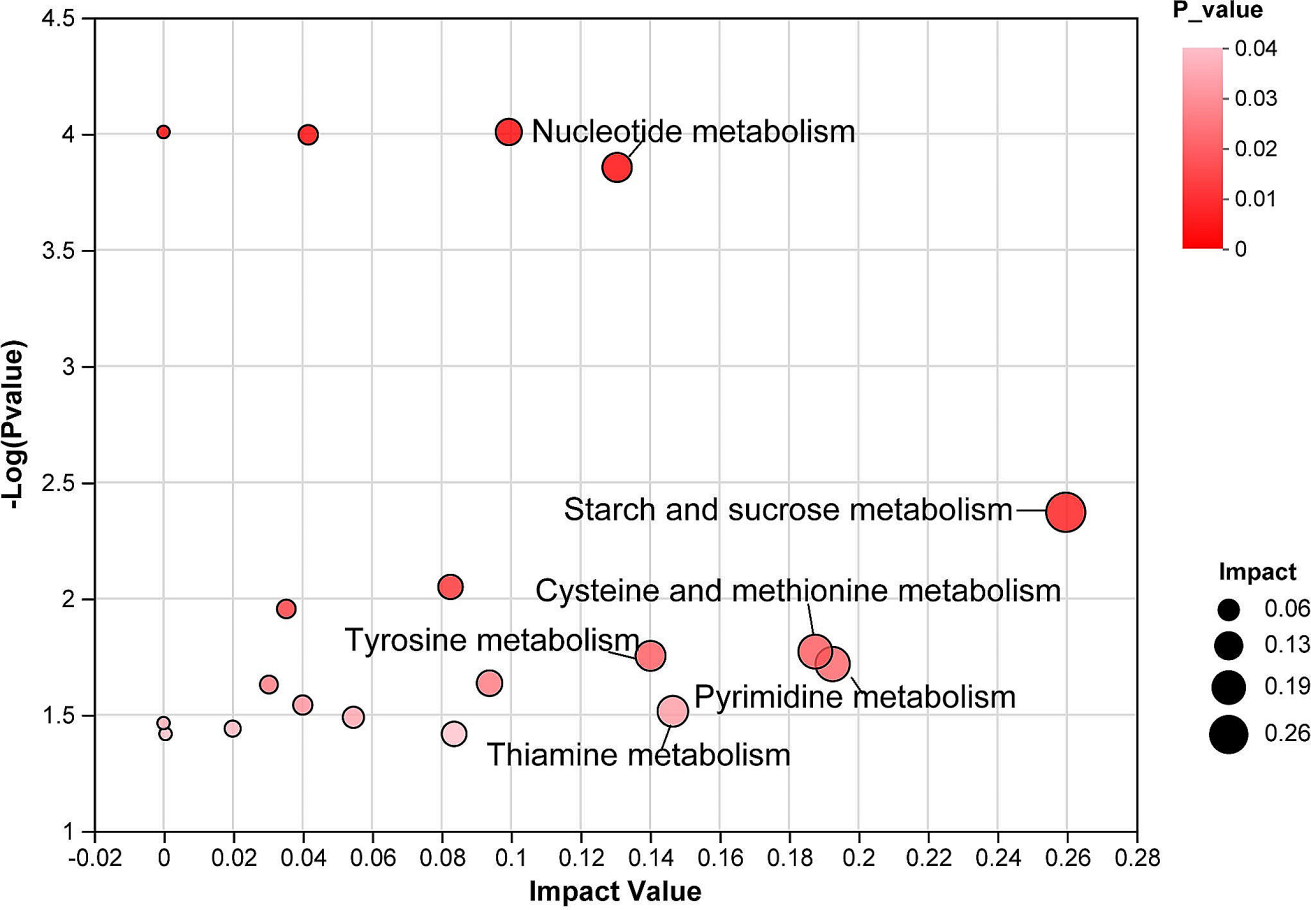
Pathway analysis of the hydrophilic and lipid metabolites in the spinal cord of PDN rats. Every single bubble in the figure represents a Kyoto Encyclopedia of Genes and Genomes pathway. The horizontal axis articulates the hierarchy of metabolites within the pathway, their ascendancy delineated by the august impact values. The vertical axis captures the significance of metabolite enrichment in the pathway, measured by the logarithm of the *P* value (−log_10_*P* value). The size of each bubble corresponds to its respective impact value

To further examine the roles of differential hydrophilic and lipid metabolites in the progression of PDN, pathway enrichment analysis was conducted using MetaboAnalyst 5.0 online. Based on the impact value and a significance threshold of *P* < 0.05, the top six metabolic pathways significantly perturbed in PDN rats were starch and sucrose metabolism (three hits: UDP-D-glucose, D-glucose 6-phosphate, D-glucose 1-phosphate), tryptophan metabolism (three hits: serotonin, L-tryptophan, quinolinic acid), pyrimidine metabolism (three hits: 2’-deoxycytidine, uridine 5’-monophosphate, uracil), cysteine and methionine metabolism (three hits: O-succinyl-L-homoserine, L-homocysteine, L-Methionine), thiamine metabolism (two hits: thiamine monophosphate, L-tyrosine), tyrosine metabolism (three hits: rosmarinic acid, L-tyrosine, fumaric acid), and nucleotide metabolism (five hits: 2’-deoxycytidine, uridine 5’-monophosphate, uracil, guanine, AMP) (Fig. 5).

### Correlation analysis between metabolites and PDN

The PWT and PWL serve as indicators of the severity of neuropathic pain in rats. Therefore, we performed a correlation analysis to examine the relationship between differential metabolites and behavioral characterization of neuropathic pain.

**Fig. 6:**
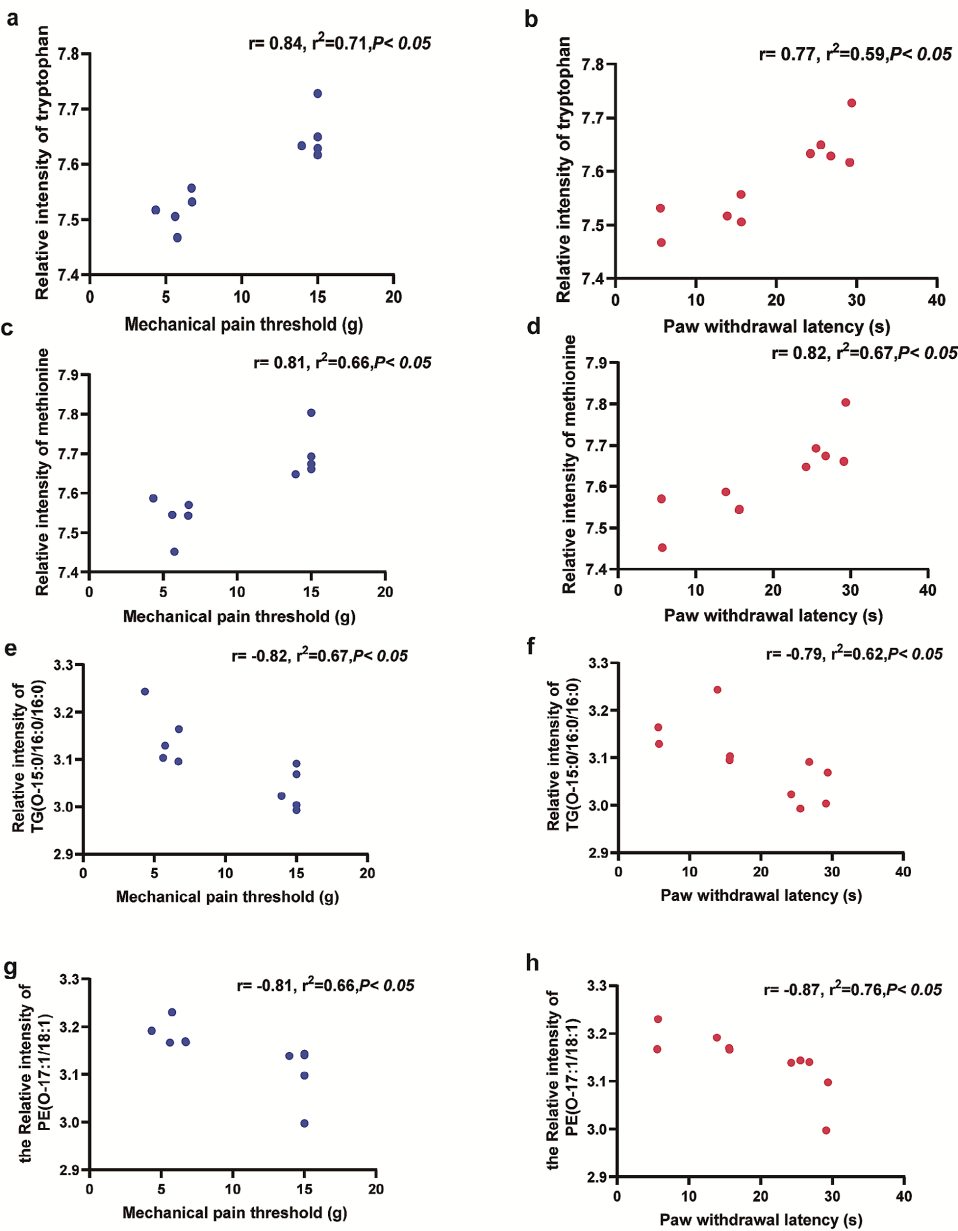
Correlation analysis results between typical differential metabolites and the PWT and PWL. **a**, **b** Correlation between tryptophan and pain behavior; **c**, **d** Correlation between methionine and pain behavior; **e**, **f** Correlation between triacylglycerol (O-15:0/16:0/16:0) and pain behavior; **g**, **h** Correlation between phosphatidylethanolamine (O-17:1/18:1) and pain behavior

Heatmaps that showed the intensity of correlations between all the significant differential metabolites and PWT and PWL were displayed in Fig. S2. The selected metabolites in Fig. 6 showed significant correlations with thermal and mechanical pain, with r squared values > 0.49. Tryptophan, and methionine in the water-soluble fraction showed a significant positive correlation with pain behavior (r values ranging from 0.77 to 0.84, *P* < 0.05), whereas triacylglycerol (TG) and phosphatidylethanolamine (PE) in the lipid-soluble fraction showed a significant negative correlation with pain behavior (r values ranging from − 0.87 to − 0.79, *P* < 0.05; Fig. 6).

## Discussion

The spinal cord plays a pivotal role in the transmissions of nociceptive signals. It functions as a vital relay station where sensory information associated with pain is processed and transmitted to the brain for further analysis. Moreover, it actively participates in the modulation of pain signals through various mechanisms, such as the release of inhibitory neurotransmitters. In essence, elucidating the intricate mechanisms underlying spinal cord involvement holds significant clinical implications. It is noteworthy that no prior investigations have explored the changes in metabolites within the spinal cord in PDN rats. Therefore, this study employed a metabolomics approach to validate the expression of metabolites in the spinal cord, aiming to identify therapeutic targets for PDN through the identification of distinctive metabolites.

The functions of amino acids encompass the realms of nutrition metabolism, intestinal health, energy steady state, immunity, and disease manifestation. Amino acids, beyond their role in synthesizing nitrogen-containing compounds, function as signaling molecules integrated into specialized networks that regulate fundamental metabolic and physiological processes (Nie et al. 2018). One study investigated the cerebral metabolism of diabetic rats and uncovered a discernible diminution of specific amino acids: tryptophan, methionine, tyrosine, threonine, phenylalanine, histidine, and lysine (Mans et al. 1987). Little attention has been devoted to unraveling amino acid metabolism in the spinal cord of rats with PDN. In our current research, we detected a decrease in tryptophan, methionine, and tyrosine levels within the spinal cord amidst the progression of PDN. This decline in amino acid abundance might potentially disrupt energy metabolism in spinal cord cells and impair central nervous system functions. Pena, M.J. associated diminished tyrosine concentrations with diabetic nephropathy (Pena et al. 2014), which aligned with our finding that the tyrosine level was decreased in the spinal cord of PDN rats. Furthermore, the non-essential amino acid homocysteine plays a significant role in neuropathy, with elevated plasma levels of cysteine observed in patients with diabetic neuropathy (Hammad et al. 2017). Homocysteine has also been independently in connection with the prevalence of diabetic neuropathy in individuals afflicted with type 2 DM (Hammad et al. 2017; Rehman et al. 2020). Therefore, it is crucial to explore in greater depth the complex mechanisms by which homocysteine influences the progression of PDN. In addition to energy metabolism, the role of amino acids and their derivatives as neurotransmitters is essential in the complex mechanisms of neuropathic pain. The amino acids (analgesic neurotransmitters), such as L-tyrosine and L-tryptophan, are downregulated within the cerebral domains of rats undergoing PDN advancement (Zhang et al. 2021). Pharmacological agents targeting these neurotransmitters, such as tricyclic antidepressants and serotonin (5-HT) and noradrenaline reuptake inhibitors, have shown efficacy in alleviating neuropathic pain and enhancing the quality of life in individuals with PDN. These medications work by increasing noradrenaline and 5-HT levels within the synaptic cleft (Dharmshaktu et al. 2012; Rastogi and Jude 2021). Recent investigations employing animal models underscored the centrality of noradrenaline in impeding neuropathic pain, its influence is mediated through the stimulation of a2-adrenergic receptors and the enhancement of the descending noradrenergic inhibitory system (Obata 2017). Activation of locus coeruleus-spinal cord noradrenergic neurons has been documented to ameliorate neuropathic pain in mice by augmenting the efflux of noradrenaline while concurrently reducing the neuroinflammatory response of astrocytes and microglia within the spinal dorsal horn (Li et al. 2022). The tryptophan–kynurenine pathway, a significant route for tryptophan metabolism in the brain, can be influenced by a low-grade inflammatory environment often associated with DM (Koziel and Urbanska 2023). Dopamine and 5-HT enhance the inhibitory effect of norepinephrine on neuropathic pain (Leventhal et al. 2007). In the current research, the attenuation in the expression levels of tyrosine and tryptophan intimates a plausible decline in noradrenaline, dopamine, and 5-HT levels within the central nervous system of rats with PDN. Hence, this decrement of tyrosine and tryptophan in the spinal cord may be postulated to wield a pivotal influence on the pathophysiology of PDN.

Additionally, the role of lipids in the development of PDN has garnered increasing attention. Beyond their function as energy storage molecules, lipids stand as indispensable components within the intricate tapestry of cell membranes, playing a pivotal role in the orchestration of cellular signal transduction (Markgraf et al. 2016). Several studies (Afshinnia et al. 2022; Doty et al. 2022; O’Brien et al. 2020; Song et al. 2022) have suggested that dysregulation of lipid metabolism is a significant factor in the onset and progression of PDN. Examining lipids may provide profound insights into the intricate mechanisms underlying neural damage in PDN.

Diglycerides (DG), which consist of two fatty acid molecules attached to a glycerol backbone, are glycerol esters that play a significant biological role in lipid metabolism and cellular signal transduction. DG appear to be an important link between tissue lipids and insulin resistance, as previous studies have revealed elevated DG concentrations in the skeletal muscles of type 2 DM patients (Bergman et al. 2012; Moro et al. 2009), suggesting a positive correlation between intramyocellular DG and insulin resistance. In coincidence with these findings, our study also elucidated a conspicuous surge in DG concentrations within the spinal cord of rats exhibiting PDN. This observation aligns with a finding that elevated DG levels distinguish between obese subjects with and without neuropathy in plasma lipidomics analysis (Guo et al. 2022). DG also serve as a precursor for the synthesis of triglycerides by the enzyme diacylglycerol O-acyltransferase 2, which is increased in the sural nerves of diabetic patients with neuropathy and the sciatic nerves of type 2 DM mice with neuropathic pain. This indicated that aberrant neural lipid signaling may be a significant factor in the peripheral neuropathy of type 2 DM (O’Brien et al. 2020). Moreover, elevated DG levels can activate the protein kinase C signaling pathway, leading to oxidative stress, inflammatory responses, and ultimately, nerve damage (Kolczynska et al. 2020; Szendroedi et al. 2014).

Compared with DG, TG are ester compounds composed of glycerol and three fatty acid molecules, serving as the primary form of energy storage. Experimental and clinical studies have shown that hypertriglyceridemia can contribute to the small fiber neuropathy progress in diabetes (Iqbal et al. 2021). In a prospective cohort study of patients with diabetes spanning 52 weeks (Wiggin et al. 2009), an association was observed between elevated TG levels and a 25% reduction in myelinated fiber density in the sural nerve. Another study investigating diabetic neuropathy using skin biopsies to evaluate intraepidermal nerve fiber density revealed a correlation between TG and the loss of small unmyelinated axons (Smith and Singleton 2013). Similarly, in our study of PDN in rats, the TG within the spinal cord predominantly exhibited an increasing trend. Although painful peripheral neuropathy is an uncommon clinical feature of an eloquent expression of primary dyslipidemia, further research is needed to explore the correlation between these factors.

In addition to lipid metabolism, this study also observed changes in structural lipids. Phosphatidylcholine (PC) and PE are vital lipid components of cell membranes, and their ratio determines membrane curvature, thereby regulating mitochondrial biogenesis and bioenergetics. Decreased PC levels signify potential alterations in mitochondrial structure, further impacting mitochondrial function (Afshinnia et al. 2022) and ultimately leading to nerve damage. Afshinnia et al. investigated the relationship between lipid levels and the subsequent development of diabetic neuropathy in individuals with type 2 DM over a decade-long period (Afshinnia et al. 2022). They observed a correlation between reduced total abundance of PC and the eventual development of neuropathy (*P* = 0.016). Patients who developed neuropathy 10 years later exhibited decreased levels of most monounsaturated and polyunsaturated PC species in their serum. Similarly, our study also delineated a synchronous diminution in PC concentration in the spinal cord. Rumora et al. exposed a discernible attenuation in plasma PC concentrations among those with type 2 DM (Rumora et al. 2021). They not only observed reduced plasma PC levels in patients with type 2 DM but also identified an increase in plasma PE, consistent with the findings of our study. A decline in the PC: PE ratio can disrupt mitochondrial function and impair ATP production (Basu Ball et al. 2018), indicating mitochondrial dysfunction in the spinal cord and potentially contributing to the mechanism of nerve damage in PDN.

Ceramides are a class of bioactive lipid molecules that serve not only as important cell membrane components but also act as signaling molecules and regulators. Abnormal levels of these molecules may be related to the genesis and progression of PDN. Through metabolomics analysis, we observed a decrease in ceramide in the spinal cord of PDN rats. This finding is consistent with the antecedent research on plasma specimens from type 2 DM patients with neuropathic manifestations, which also showed reduced ceramide levels (Xu et al. 2023). Concomitantly, an independent investigation discerned a reduction in ceramide abundance within the plasma of type 2 DM patients (Rumora et al. 2021). Significantly, a clinical study of type 1 DM propounded the prospective utility of plasma ceramide quantification as diagnostic and prognostic indices for diabetic neuropathy (Hammad et al. 2017). Considering the pivotal role of ceramides in sphingolipid metabolism, this finding also implied that modulation of sphingolipid metabolism may serve as a novel therapeutic strategy for type 2 DM patients with PDN.

In conclusion, abnormal metabolomics and lipid metabolism can lead to structural and functional damage of neuronal membranes, increased inflammatory responses, and oxidative stress, thereby promoting the development of diabetic neuropathy. Therefore, regulation of differential metabolites may be an important strategy to prevent and treat diabetic neuropathy.

### Electronic supplementary material

Below is the link to the electronic supplementary material.

Supplementary Material 1

Supplementary Material 2

Supplementary Material 3

## Data Availability

No datasets were generated or analysed during the current study.
