# Supplementary material for: Metabolomic and lipidomic profiling of the spinal cord in type 2 diabetes mellitus rats with painful neuropathy
Source: Metab Brain Dis. 2024 Jul 9;39(6):1117–30. doi: 10.1007/s11011-024-01376-x (PMC11349861; doi:10.1007/s11011-024-01376-x)
Supplement: Supplementary file 3 — Supplementary Material 3 [file 11011_2024_1376_MOESM3_ESM.docx]

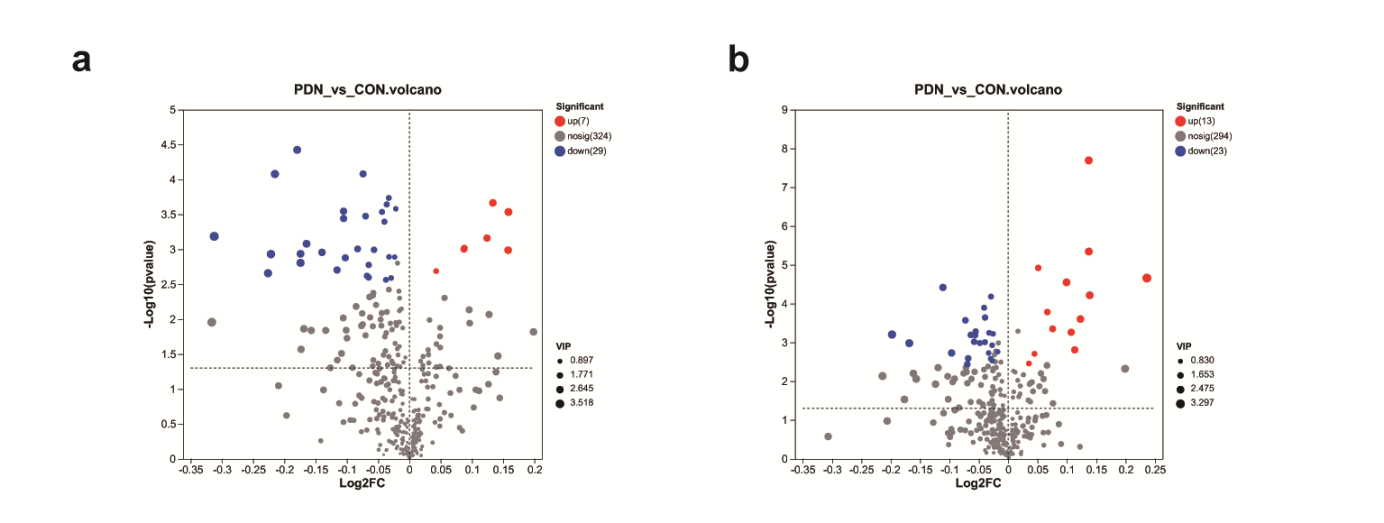


Supplement Figure 1. Volcano plot of hydrophilic metabolites in both positive and negative ion mode.

a: Positive ion mode of hydrophilic substances; b: Negative ion mode of hydrophilic substances.

Every datum on this graphical tableau aligns with a metabolite, where the red unveils the significantly upregulated entities, the blue unveils the significantly downregulated counterparts, and the grey unveils those metabolites that remain non-significantly different from the normal group. The classification is based on a VIP > 1 and a significance level of false discovery rate < 0.05.


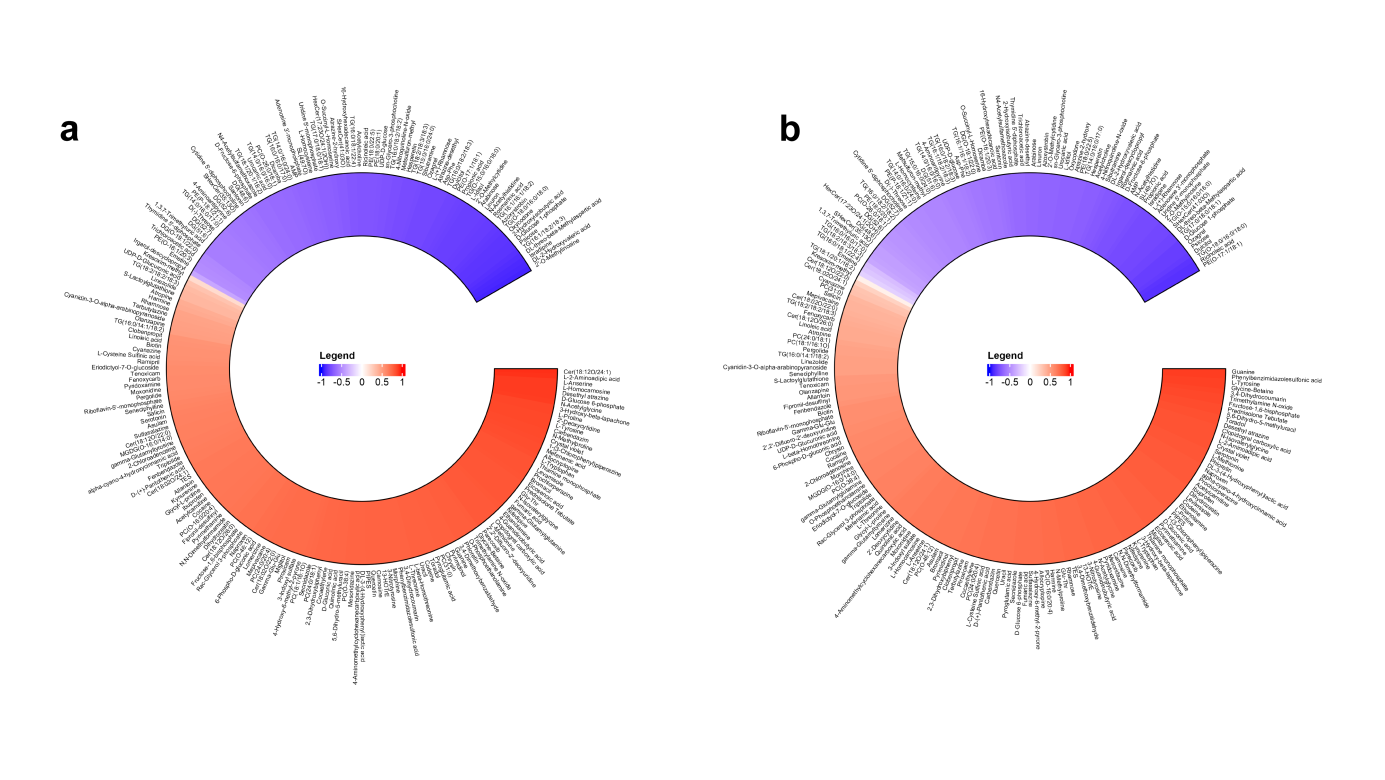


Supplement Figure 2. Correlation analysis results between all differential metabolites and the PWT and PWL.

a: Correlation between differential metabolites and the PWT; b: Correlation between differential metabolites and PWL.

Red indicates a positive correlation between metabolites and pain behavior, and purple indicates a negative correlation between metabolites and pain behavior; the deeper the color, the stronger the correlation.
